# Grain quality in superior and inferior grains of soft and non-soft rice varieties from the Yangtze River Delta

**DOI:** 10.3389/fpls.2025.1562708

**Published:** 2025-03-11

**Authors:** Xi Chen, Jiale Cao, Zhongtao Ma, Jianghui Yu, Ying Zhu, Fangfu Xu, Qun Hu, Guodong Liu, Guangyan Li, Haiyan Wei

**Affiliations:** ^1^ Jiangsu Key Laboratory of Crop Genetics and Physiology/Jiangsu Key Laboratory of Crop Cultivation and Physiology, Agricultural College of Yangzhou University, Yangzhou, China; ^2^ Jiangsu Co-Innovation Center for Modern Production Technology of Grain Crops, Yangzhou University, Yangzhou, China; ^3^ Research Institute of Rice Industrial Engineering Technology, Yangzhou University, Yangzhou, China

**Keywords:** soft rice, eating quality, appearance quality, starch, protein, pasting properties

## Abstract

**Objective:**

This study aims to establish a scientific basis for improving rice quality by investigating the relationship between variations in eating and appearance quality and the starch and protein content in soft and non-soft rice varieties.

**Methods:**

Two soft rice varieties, Nanjing 5718 and Nanjing 9108, were compared with two non-soft rice varieties, Huaidao 5 hao and Huajing 5 hao. The study focused on eating and appearance quality, starch and protein content, and how these factors varied between superior and inferior grains within each variety.

**Results:**

Compared to non-soft rice, soft rice has some disadvantages in appearance quality, which is characterized by high chalky grain rate and chalkiness degree, and large differences in appearance between superior and inferior grains. This is mainly due to the low proportion of high grain weight grains, low amylose content, abnormal development of starch structure, and large protein bodies with high globulin and glutenin content, which destroys the close accumulation of starch particles, resulting in increased light scattering, increased chalkiness, and reduced transparency. Although the appearance quality of soft rice is not outstanding, its eating quality is extremely excellent, which is embodied in good appearance, high viscosity, good balance, high taste value and low hardness. This superior performance may be due to the high proportion of amylopectin in soft rice, which helps to improve the overall taste. In contrast, the amylose content of non-soft rice is higher, which leads to the increase of rice hardness and the decrease of viscosity. In addition, the gelatinization property of soft rice starch is more favorable, which makes the starch dissolve more in the cooking process, so as to further improve its eating quality.

## 1 Introduction

China is the country with the largest planting area and the highest total yield of japonica rice in the world (Cao et al., 2024). Within China, the Yangtze River Delta (YRD) stands out as a dominant japonica rice production region, characterized by exceptional yield per unit area and substantial total output. This makes the YRD crucial for ensuring regional and national food security (Zhu, 2022; Ma et al., 2024). However, excessive application of nitrogen fertilizer, coupled with the high temperature and humidity environment in the area, has led to an increase in protein content, hardness, and chalkiness, which in turn has resulted in a decline in rice quality (Lin et al., 2016; Zhu, 2022). Although the Yangtze River Delta region is renowned for its high rice yields (Zhu et al., 2021a; Zhu et al., 2021b), achieving a balance between high productivity and high quality remains a significant challenge globally. Advances in plant breeding have led to the development of soft rice varieties, which are highly valued for their soft texture, good elasticity, and excellent eating quality (Dou et al., 2017; Zhang et al., 2019; Shi et al., 2022). Compared to non-soft rice, soft rice typically exhibits a translucent or cloudy appearance, which notably diminishes its appearance quality and greatly impacts its market value (Zhu, 2022). As a result, the enhancement of appearance quality in soft rice varieties, while simultaneously maintaining their favorable taste characteristics and high yield potential, has emerged as a pivotal objective that necessitates immediate attention.

In order to achieve a balance between high yield and high quality in rice cultivation in the Yangtze River Delta region, it is crucial to explore the impact of spikelet position on rice quality (Peng et al., 2015). Studies have demonstrated that variations in spikelet position leads to significant differences in rice quality (Jiang et al., 2022). Specifically, superior grains positioned at the apex of the spikelet tend to attain fullness earlier, resulting in higher plumpness and overall superior quality (Yin et al., 2013). These superior grains exhibit excellent processing qualities, such as higher quality of brown rice rate, milled rice rate, and head rice rate, as well as enhanced appearance quality with notably lower chalkiness and chalkiness degree (Yin et al., 2013; Li et al., 2022). Additionally, superior grains possess higher palatability, elasticity, and balance. Conversely, inferior grains located at the base of the spikelet exhibit delayed filling, leading to reduced plumpness and quality (Jiang et al., 2022). This discrepancy is not solely reflected in rice quality but further impacts the chemical composition of rice. As the primary components of endosperm, the content and composition of starch and protein are also influenced by grain position. Superior grains typically contain higher amylose and lower protein, which are intimately associated with spikelet position (You et al., 2016). Given the intricate interactions among floret position, rice quality, and starch-binding protein complexes, further research is essential to elucidate the underlying mechanisms responsible for this phenomenon and to inform strategies aimed at enhancing rice quality. At present, a large number of studies have focused on the formation of superior and inferior granules and their pairing (Liu et al., 2017; Wei et al., 2018; Chen et al., 2021). However, these studies have predominantly concentrated on a single grain type, with limited research exploring the quality differences between various types of superior and inferior grains. Therefore, in this study, we selected two soft rice varieties (Nanjing 5718 and Nanjing 9108) and two non-soft rice varieties (Huaidao 5 hao and Huajing 5 hao), which are widely cultivated in the Yangtze River Delta region (Zhu, 2022), to construct a scientific framework for optimizing rice quality by comparatively analyzing the quality disparities between these two types of varieties in terms of superior and inferior grains, as well as their interactions with starch and protein contents. The objective of this study is not only to enhance the overall quality and market competitiveness of japonica rice in the Yangtze River Delta region and globally but also to deepen our understanding of the relationship between starch and protein composition and rice quality, thereby providing novel strategies and methodologies for improving rice quality.

## 2 Materials and methods

### 2.1 Materials

#### 2.1.1 Plant material

Soft rice varieties (Nanjing 5718 and Nanjing 9108) and non-soft rice varieties (Huaidao 5 hao and Huajing 5 hao), primarily promoted and cultivated in the Yangtze River Delta region, were utilized as materials in this study. Field experiments were conducted on a research farm belonging to Yangzhou University, located in Jiangsu Province, China (32°30’N, 119°25’E), during the rice growing season (May–October) of 2022 and 2023.

#### 2.1.2 Field management

The experimental site was selected based on plots characterized by moderate to high soil fertility and convenient irrigation facilities. A randomized block design was employed. The cultivation method involved machine-transplanted blanket seedlings, which were spaced in rows 30 cm apart with 12 cm intervals between holes, accommodating 4-5 plants per hole. The nitrogen fertilizer was applied at a rate of 270 kg/ha, with a distribution ratio of base fertilizer, tiller fertilizer, and spike fertilizer set at 35:35:30. Tiller fertilizer was applied seven days post-transplanting, while spike fertilizer was applied at the reversed four-leaf stage. The nutrient ratio of N: P: K was upheld at 2:1:2, with phosphate delivered as a singular basal fertilizer application and potash evenly distributed before plowing and at the jointing phase. Water management, pest and weed management, and other pertinent cultivation practices were implemented in accordance with high-yield cultivation standards.

### 2.2 Methods

The mature samples were then separated into superior grains (SG), which are located at the apical of the primary branches, and inferior grains (IG), which are located on proximal secondary branches. And used for the determination of the following indicators.

#### 2.2.1 Processing quality

According to GB/T 17891-2017 (2018), brown rice rate, milled rice rate, and head rice rate were determined.

#### 2.2.2 Appearance quality

The chalkiness of the seeds was determined according to GB/T 17891-2017 (2018). Subsequently, the chalky rate and chalkiness were computed using the methodology specified by WS-SC-E in China. The length, width, thickness, and length-width ratio of brown rice were measured using an electronic digital caliper. The volume (V) of a single grain was calculated using method by Jain and Bal (1997), as expressed by the formula: $V=0.25*\left(\frac{\pi }{6}\right)*L*(W+T{)}^{2}$ , where L is the grain length, W is the width, and T is the thickness. Brown rice plumpness= $\frac{Vbrown\;rice}{Vpaddy}*100\%$ . The transparency of the rice was quantified by its transmittance when the rice sample was contained in a 1 cm thick cuvette, using a colorimeter equipped with a D65 light source (CM-5, Konica Minolta, Tokyo, Japan). The transparency of mature normal seeds and non-chalky seeds was assessed in this manner.

#### 2.2.3 Scanning electron microscopy

Following the method described by Xi et al. (2014), scanning electron microscopy (SEM) (Gemini SEM 300, Carl Zeiss, Oberkochen, Germany) was used to examine the microscopic morphology of rice endosperm and starch samples. The ends of the rice grains were trimmed using a single-edged blade to expose a cross-section approximately 2 mm thick, which was then secured to the sample stage with double-sided tape. Subsequently, the cross-section was coated with a thin layer of gold and observed under a 5 KV acceleration voltage at a magnification of 1200x.

#### 2.2.4 Percentage of high-weight and low- weight grains

Following the method described by Cao et al. (2024). Based on the average weight of superior and inferior grains at maturity, the grains were categorized into high-weight and low-weight groups. High-weight grains were identified as those with an individual weight equal to or greater than the average weight of the superior grains. Conversely, low-weight grains were classified as those having an individual weight less than or equal to the average weight of the inferior grains. The percentage of high-weight or low-weight grains was determined by dividing the number of high-weight or low-weight grains by the total number of spikelet per panicle.

#### 2.2.5 Eating quality

Following the method described by Zhu (2022), the appearance and taste value of cooked rice were evaluated using a taste analyzer (STA1A, SATAKE, Japan), selecting ‘Japanese japonica rice’ as the preset detection line.

#### 2.2.6 Starch, protein and their components

AC was determined by the iodine adsorption method (Tan et al., 1999). Total starch content was measured using a total starch assay kit (Megazyme, Bray, Ireland), following the manufacturer’s instructions. Amylopectin content was calculated using the equation: amylopectin content = total starch content - amylopectin content. PC was assessed through the Kjeldahl method, utilizing an automatic Kjeldahl apparatus (Kjeltec 8200, Foss, Hillerød, Denmark). The protein components were analyzed according to the method described by Christine V. Sapan et al. (1999) and Zhu (2022). Rice flour (0.2 g) in a 10-mL centrifuge tube was oscillated with 2 mL ultrapure water for 2 min before centrifuging at 2000g for 15 min, and the supernatant which contained the albumin was collected. To fully extract as much of the remaining albumin as possible from the precipitate, the precipitate was mixed with 2 mL ultrapure water, oscillated, and centrifuged to collect the supernatant three times. The first extraction of albumin and any remaining extracted albumin were pooled together. The precipitate remaining after the albumin extraction was mixed with 2 mL 50 g L^−1^ NaCl solution to extract globulin by following the same process used to collect the albumin. The precipitate, after completing the globulin extraction, was mixed with 2 mL 700 mL L^−1^ ethyl alcohol solution, oscillated for 2 min, then oscillated in an 80°C water bath for 30 min with two small glass beads in a sealed tube, and then oscillated for two more minutes before centrifuging at 2000g for 15 min to obtain the supernatant containing prolamin. This prolamin extraction process was repeated three time to extract any remaining prolamin. The precipitate after the prolamin extraction was mixed with 2 mL of 2.004 g L^−1^ NaOH solution to extract the glutelin by following the same process used to collect the albumin and globulin.

#### 2.2.7 Pasting properties

The pasting properties of rice flour were measured using a Rapid Viscosity Analyzer (RVA Tec Master, Perten, Sweden), following the methodology described by Zhu (2022).

### 2.3 Data processing and analysis

The data were processed using Microsoft Excel 2019. Statistical analysis was performed using SPSS 27 software (IBM Corporation, Armonk, NY, USA), and the significance test was conducted using the least significant difference method (LSD) with a significance level set at p< 0.05. Graphical representations were generated using Origin 2021 (Origin Lab, Northampton, MA, USA). The results obtained from the two-year experimental dataset did not exhibit statistical significance, and the inter-year variation was found to be not significant. Therefore, the average value from the two years was considered for further analysis.

## 3 Results and analysis

### 3.1 Processing quality

Analysis of processing quality revealed differences between soft and non-soft rice (**Table 1**). Soft rice exhibited a lower brown rice rate compared to non-soft rice, with reductions of 0.28% to 1.66% in superior grains and 0.53% to 0.93% in inferior grains. However, no statistically significant differences were observed in milled rice rate or head rice rate between the two groups. Notably, within each variety, inferior grains demonstrated lower processing efficiency compared to superior grains, a trend that was more pronounced in soft rice. Specifically, inferior grains of soft rice showed decreased brown rice rate of 1.36% to 2.63%, decreased milled rice rate of 1.98% to 4.31%, and decreased head rice rate of 4.24% to 6.19% compared to their superior grain counterparts. Similarly, inferior grains of non-soft rice also exhibited decreased processing performance, with brown rice rate reductions of 2.00% to 2.48%, milled rice rate reductions of 1.63% to 3.71%, and head rice rate reductions of 4.46% to 5.92% compared to their respective superior grains.

**Table 1:** Differences in processing quality of superior and inferior grains between soft and non-soft rice (%).

Cultivar	Grain position	Brown rice rate	Milled rice rate	Head rice rate
NJ5718	SG	84.26±0.71a	73.73±1.25ab	67.43±0.95a
	IG	82.04±0.64c	71.43±0.64c	64.57±0.6b
NJ9108	SG	83.35±0.88b	74.65±0.61a	68.38±0.88a
	IG	82.22±0.63c	72.27±0.63bc	64.15±0.51b
HD5	SG	84.76±1.29a	74.05±0.33a	67.96±0.78a
	IG	82.81±1.14bc	72.32±0.73bc	63.94±1.06b
HJ5	SG	84.50±1.29a	73.52±1.19ab	67.72±1.29a
	IG	82.66±0.83bc	71.30±0.83c	64.70±0.77b
F-value	Type (T)	**	NS	NS
	Position (P)	**	**	**
	T×P	NS	NS	NS

Values in the same column with different letters are significantly different (p < 0.05). SG, superior grains; IG, inferior grains. Type represents two types of soft rice and non-soft rice. Position represents the position of superior and inferior grains. NS, No significant correlation. **p<0.01.

### 3.2 Appearance quality traits

#### 3.2.1 Chalkiness and transparency

Analysis of appearance quality in superior and inferior grains of both soft and non-soft rice revealed substantial differences in chalkiness characteristics, transparency, and grain traits (**Table 2**). Compared to non-soft rice, superior grains of soft rice exhibited a significantly higher chalky grain rate, increase by 80.05% to 115.94%. Additionally, the degree of chalkiness was elevated by 410.90% to 483.80%, while their transparency was reduced by 15.27% to 26.86%. In inferior grains of soft rice, these differences were even more apparent, with chalky grain rate increases of 70.44% to 89.89% and chalkiness degree increases of 118.36% to 140.07%. Concurrently, transparency decreased more significantly, by 30.45% to 36.37%. Notably, within the soft rice category, inferior grains exhibited a chalky grain rate that was 206.24% to 250.99% higher, a chalkiness degree that was increased by 771.51% to 844.70%, and a transparency that was 39.92% to 47.30% lower than that of superior grains. The differences in chalky grain rate, chalkiness degree, and transparency between superior and inferior grains of soft rice were 41.18, 27.44, and 4.96, respectively. In non-soft rice, inferior grains exhibited increased chalkiness and reduced transparency compared to superior grains. Specifically, the chalky grain rate was 232.80% - 287.99% higher, the chalkiness degree was 1910.47% to 2230.00% greater, and the transparency was 29.82% to 36.83% lower in inferior grains. The differences in chalky rate, chalkiness, and transparency between superior and inferior grains of non-soft rice were 24.44, 12.90, and 4.95, respectively.

**Table 2:** Differences in appearance quality of superior and inferior grains between soft and non-soft rice .

Cultivar	Grain position	Chalky grain rate (%)	Chalkiness degree (%)	Transparency (%)	Grain length (mm)	Grain width (mm)	Grain thickness (mm)	Length/width ratio	Volume	Brown rice plumpness (%)
NJ5718	SG	18.33±0.22e	3.25±0.11c	10.48±0.28b	5.27±0.06a	3.20±0.06a	2.25±0.05a	1.64±0.02de	20.49±0.05a	52.50±1.32c
	IG	57.58±1.25a	30.68±0.78a	5.91±0.30d	5.02±0.07c	3.08±0.04b	2.14±0.02c	1.61±0.03e	17.89±0.12b	51.81±0.65c
NJ9108	SG	16.41±0.28f	3.38±0.14c	9.83±0.32c	4.79±0.03d	2.85±0.07d	2.14±0.02c	1.68±0.03cd	15.57±0.28c	58.28±0.43b
	IG	56.13±1.74b	29.44±0.52a	5.52±0.35d	4.64±0.10e	2.73±0.04e	1.99±0.06e	1.70±0.03d	13.52±0.20e	58.24±0.76b
HD5	SG	9.11±0.12g	0.64±0.02d	12.37±0.43ab	5.19±0.04b	2.99±0.08c	2.17±0.02b	1.76±0.04b	18.10±0.15b	59.71±0.21a
	IG	32.93±0.71d	13.48±0.57b	8.68±0.16c	4.98±0.08c	2.77±0.07e	2.08±0.03d	1.80±0.03ab	15.34±0.12c	58.97±0.54b
HJ5	SG	8.49±0.18g	0.58±0.02d	13.44±0.18a	5.17±0.13b	2.98±0.07c	2.18±0.03b	1.74±0.03b	17.97±0.35b	58.74±0.43b
	IG	30.32±0.52c	12.78±0.46b	8.49±0.14c	4.95±0.10c	2.74±0.07e	2.07±0.04d	1.81±0.02a	14.96±0.14d	58.52±0.33b
F-value	Type (T)	**	**	**	**	**	**	**	**	**
	Position (P)	**	**	**	**	**	NS	**	NS	NS
	T×P	**	**	NS	NS	*	**	NS	**	**

Values in the same column with different letters are significantly different (p < 0.05). SG, superior grains; IG, inferior grains. Type represents two types of soft rice and non-soft rice. Position represents the position of superior and inferior grains. NS, No significant correlation, *p<0.05, **p<0.01.

#### 3.2.2 Grain shape

While overall grain shape differences were not substantial, subtle variations were observed in the length/width ratio and brown rice plumpness (**Table 2**). Compared to non-soft rice, both superior and inferior grains of soft rice exhibited reductions in the length/width ratio, with decreases of 3.24% to 6.74% for superior grains and 5.63% to 10.70% for inferior grains. Similarly, reductions in plumpness were observed, with decreases of 9.86% to 11.10% for superior grains and 0.39% to 1.99% for inferior grains. Specifically, inferior grains of soft rice demonstrated decreases in grain length (4.62% - 12.10%), grain width (7.62% - 14.79%), grain thickness (0.05% - 11.52%), length/width ratio (3.15% - 5.64%), and volume (12.97% - 34.11%). The differences between superior and inferior grains of soft rice in terms of grain length, width, thickness, length/width ratio, and volume were 0.64, 0.47, 0.26, 0.10, and 7.00, respectively. Similarly, for non-soft rice, inferior grains showed reductions in grain length (3.60% - 4.61%), grain width (6.92% - 8.39%), grain thickness (4.37% - 5.05%), length/width ratio (2.06% - 3.86%), and volume (14.65% - 17.33%), accompanied by a slight decrease in plumpness (0.41% - 0.85%) compared with superior grains. The differences between superior and inferior grains of non-soft rice in grain length, width, thickness, length/width ratio, volume, and brown rice plumpness were 0.24, 0.25, 0.11, 0.07, 3.14, and 0.5, respectively.

#### 3.2.3 Scanning electron microscope

**Figure 1:**
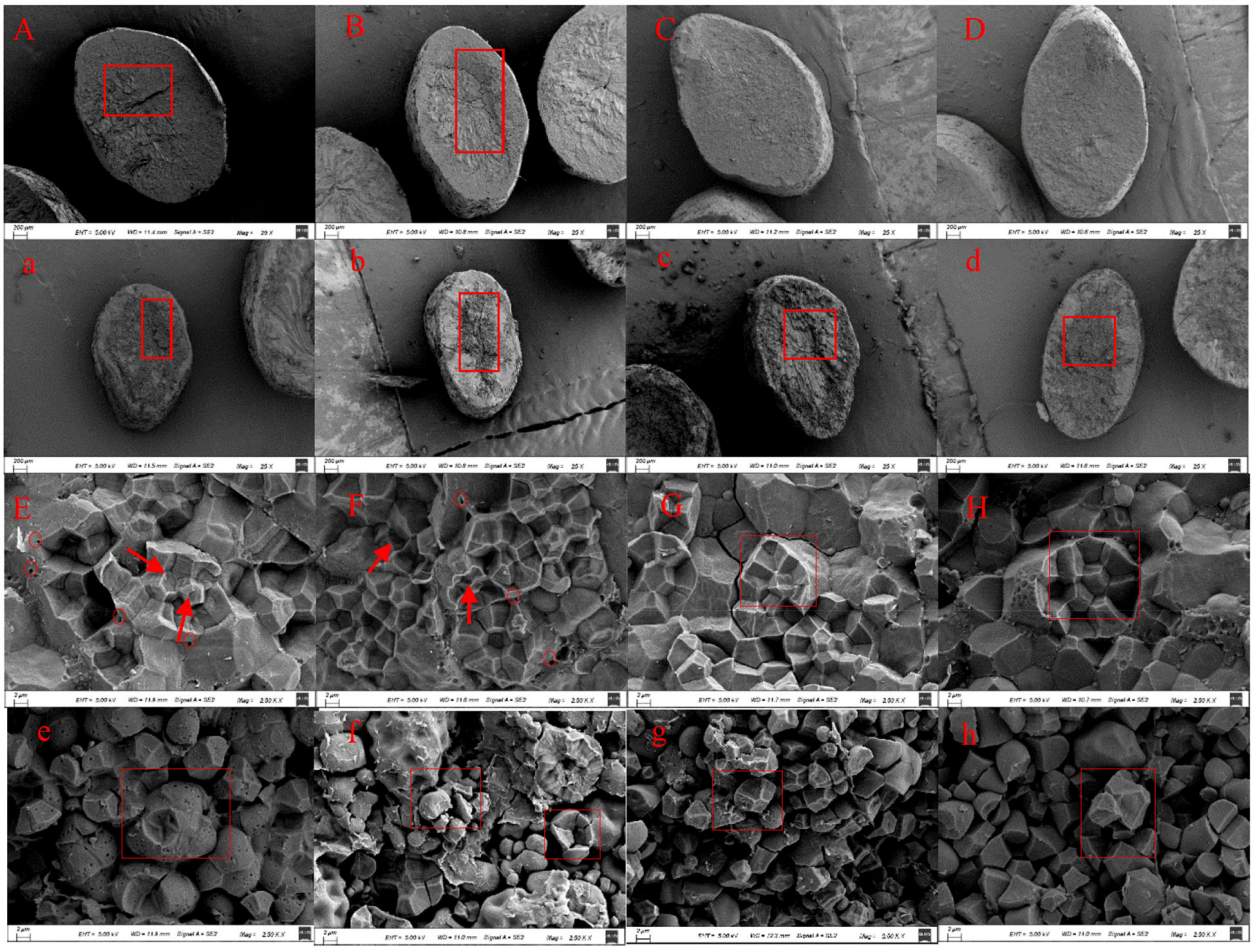
Morphology observation of mature grains of superior and inferior grains of soft and non-soft rice (×25, ×2500). **(A, E)** were NJ5718 superior grains, **(B, F)** were NJ9108 superior grains, **(C, G)** were HD5 superior grains, **(D, H)** were HJ5 superior grains, **(a, e)** were NJ5718 inferior grains, **(b, f)** were NJ9108 inferior grains, **(c, h)** were HD5 inferior grains, **(d, h)** were HJ5 inferior grains.

Microscopic analysis of endosperm transverse sections provided insights into the structural basis of these appearance differences. Compared to non-soft rice, superior grains of soft rice exhibited a noticeably loose endosperm structure characterized by prominent cracks (**Figures 1A-D**), which likely contributes to the higher chalkiness. Conversely, inferior grains of soft rice exhibited even greater collapse and a higher number of cracks, predominantly located within the intergranular spaces of composite starch granules (**Figures 1d-g**). This suggests a structural weakness and potentially greater susceptibility to disintegration. Further analysis of endosperm starch granules (**Figure 1**) revealed that, compared to non-soft rice, superior grains of soft rice exhibited starch granules with signs of collapse and cavities (**Figures 1E-H**), potentially indicating incomplete starch development. In contrast, inferior grains of soft rice displayed, spherical starch granules that were loosely distributed within a loosely arranged intergranular matrix, rendering them more prone to dispersion upon extrusion (**Figures 1e-h**).

#### 3.2.4 Range and Percentage of high-weight grains and low-weight grains

Differences in the proportions of high-weight grains and low-weight grains were also observed between soft and non-soft rice (**Table 3**). Specifically, compared to non-soft rice, the proportion of high-weight grains in soft rice was reduced by 13.12% - 26.18%, whereas the proportion of low-weight grains increased by 7.64% - 11.57%.

**Table 3:** The grain weight range and quantity proportion of high-weight and low-weight grains of soft rice and non-soft rice.

Cultivar	Types	Grains weight range (mg)	Percentage (%)
NJ5718	high-weight grains	≥30.53	22.08±0.12c
	low-weight grains	≤20.94	18.89±0.17d
NJ9108	high-weight grains	≥25.18	22.97±0.09c
	low-weight grains	≤18.48	19.29±0.21d
HD5	high-weight grains	≥28.76	29.91±0.15a
	low-weight grains	≤18.89	17.55±0.87e
HJ5	high-weight grains	≥28.95	26.44±0.45b
	low-weight grains	≤19.46	17.29±0.18e

Values in the same column with different letters are significantly different (p < 0.05).

### 3.3 Eating quality

The analysis of eating quality characteristics revealed significant differences between soft and non-soft rice (**Table 4**). Compared to non-soft rice, soft rice varieties exhibited notably higher scores for appearance, viscosity, balance, and taste value, in both superior and inferior grains. Specifically, superior grains of soft rice showed a 21.77%-34.54% higher appearance score, 11.48%-24.63% greater viscosity, 62.26%-75.05% higher balance, and a 21.83%-33.70% improvement in taste value compared to superior grains of non-soft rice. Conversely, the hardness of superior grains in soft rice was 11.48%-24.63% lower than that of non-soft rice. A similar trend was observed in inferior grains, where soft rice displayed a 26.72%-39.30% higher appearance score, 27.73%-41.48% greater viscosity, 64.33%-80.29% higher balance, and a 19.51%-24.32% improvement in taste value, along with 10.66%-23.98% lower hardness values.

**Table 4:** Differences in eating quality of superior and inferior grains between soft and non-soft rice (%).

Cultivar	Grain position	Appearance	Hardness (g)	Viscosity	Balance	Taste value
NJ5718	SG	7.83±0.06c	6.17±0.05f	7.97±0.17b	8.6±0.07b	80.37±0.28b
	IG	7.73±0.07c	6.37±0.06e	7.83±0.06b	8.43±0.06c	79.27±0.78b
NJ9108	SG	8.57±0.14a	5.60±0.09h	8.70±0.00a	8.70±0.08a	85.53±0.95a
	IG	8.40±0.08b	5.80±0.10g	8.63±0.06a	8.60±0.10b	80.40±0.62b
HD5	SG	6.37±0.15d	7.43±0.05b	6.27±0.05c	4.97±0.06f	63.97±1.39e
	IG	6.03±0.06e	7.63±0.07a	6.10±0.00d	4.77±0.05g	64.67±0.15de
HJ5	SG	6.43±0.12d	6.97±0.08d	6.23±0.12cd	5.30±0.10d	65.97±1.26cd
	IG	6.10±0.10e	7.13±0.07c	6.13±0.05cd	5.13±0.05e	66.33±1.03c
F-value	Type (T)	**	**	**	**	**
	Position (P)	**	**	**	**	**
	T×P	*	NS	NS	NS	NS

Values in the same column with different letters are significantly different (p < 0.05). SG, superior grains; IG, inferior grains. Type represents two types of soft rice and non-soft rice. Position represents the position of superior and inferior grains. NS, No significant correlation, *p<0.05, **p<0.01.

Interestingly, within each variety, inferior grains generally exhibited lower taste value scores compared to superior grains. However, the differences between superior and inferior grains in the same variety were comparatively small, particularly for soft rice varieties. For instance, the appearance, viscosity, balance, and taste value scores of inferior grains in the soft rice variety NJ5718 were only 1.28%, 1.79%, 2.02%, and 1.37% lower than those of superior grains, respectively, with a corresponding 3.24% increase in hardness. Similarly, the same parameters in NJ9108 were 1.98%, 0.81%, 1.16%, and 6.00% lower, with a 3.57% increase in hardness. Non-soft rice varieties showed similar trends, with inferior grains having slightly lower scores for appearance, viscosity, balance, and taste value (5.34%, 2.79%, 4.19%, and 1.08% lower in HD5, and 5.13%, 1.63%, 3.31%, and 0.54% lower in HJ5, respectively), and higher hardness (2.69% in HD5, and 2.30% in HJ5).

### 3.4 Starch and protein content and composition

#### 3.4.1 Starch content and morphology

Analysis of starch content revealed significant differences between superior and inferior grains within both soft and non-soft rice (**Table 5**). Compared to non-soft rice, superior grains of soft rice exhibited a reduction in total starch content (TSC) by 1.83% to 2.46%, a decrease in amylose content (AC) by 40.97% to 44.82%, and an increase in amylopectin content (AP) by 8.68% to 10.80%. Similarly, inferior grains of soft rice showed a decrease in TSC by 2.64% to 3.07% and in AC by 36.58% to 39.36%, accompanied by a corresponding increase in AP by 5.10% to 5.32%. Notably, within soft rice, inferior grains had a higher AC compared to their superior grains, with the increase being between 5.55% and 13.98%. They also had a higher TSC, with an increase of 0.26% to 0.61%. However, they exhibited a lower AP, with a reduction of 0.46% to 1.53%. In contrast, inferior grains of non-soft rice had a reduced AC, with a decrease of 16.25% to 17.73%, and a higher AP, with an increase of 4.77% to 5.91%.

**Table 5:** Differences in starch content and protein content between superior and inferior grains of soft rice and non-soft rice (%).

Cultivar	Grain position	Total starch content	Amylose content	Amylopectin content	Total protein	Albumin	Globulin	Prolamin	Glutelin
NJ5718	SG	79.05±0.71b	10.09±0.10d	68.96±0.61ab	8.93±0.05bc	0.39±0.10cd	0.41±0.04d	0.43±0.10e	6.89±0.73c
	IG	78.57±0.65b	9.23±0.22f	69.34±0.57a	9.35±0.06a	0.43±0.08b	0.46±0.04c	0.45±0.08d	7.15±0.14ab
NJ9108	SG	79.02±0.29b	10.73±0.13d	68.29±0.18b	8.99±0.03b	0.40±0.07c	0.49±0.02b	0.39±0.03f	7.02±0.92b
	IG	78.82±0.27b	9.54±0.13e	69.28±0.27a	9.43±0.03a	0.49±0.08a	0.53±0.08a	0.40±0.05f	7.27±0.28a
HD5	SG	80.52±0.51a	18.28±0.19a	62.24±0.69d	8.32±0.03d	0.33±0.03e	0.38±0.08e	0.44±0.08de	6.09±1.00e
	IG	80.95±0.34a	15.04±0.13b	65.91±0.23c	8.73±0.08c	0.40±0.02cd	0.41±0.09d	0.47±0.11c	6.24±0.46de
HJ5	SG	81.02±0.55a	18.17±0.10a	62.84±0.61d	8.28±0.10d	0.31±0.24e	0.33±0.04f	0.50±0.07b	5.98±0.83f
	IG	81.06±0.25a	15.22±0.12b	65.84±0.31c	8.69±0.06c	0.38±0.20d	0.39±0.01e	0.53±0.02a	6.30±0.18d
F-value	Type (T)	**	**	**	**	**	**	**	**
	Position (P)	NS	**	**	**	**	*	NS	**
	T×P	NS	**	**	NS	NS	NS	NS	NS

Values in the same column with different letters are significantly different (p < 0.05). SG, superior grains; IG, inferior grains. Type represents two types of soft rice and non-soft rice. Position represents the position of superior and inferior grains. NS, No significant correlation, *p<0.05, **p<0.01.

**Figure 2:**
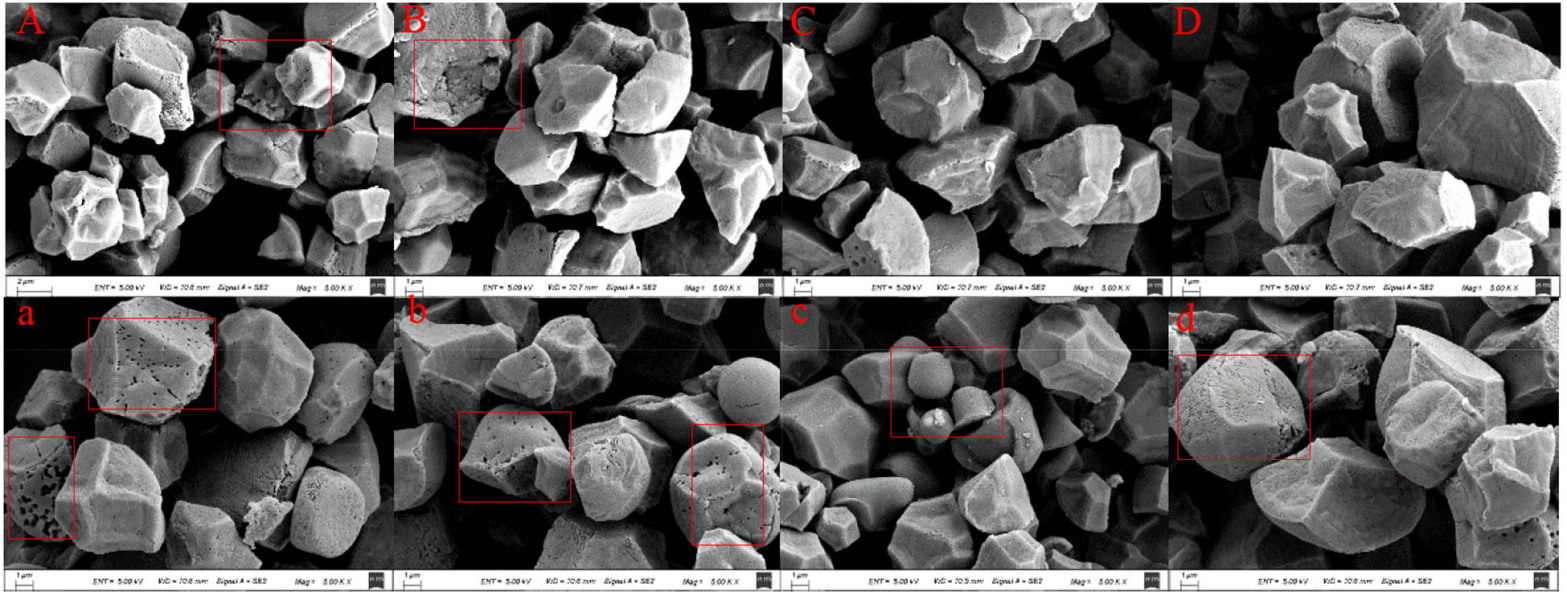
Scanning electron microscope observation of starch at different grain positions of soft rice and non-soft rice. **(A, a)** were NJ5718, **(B, b)** were NJ9108, **(C, c)** were HD5, **(D, d)** were HJ5; Capital letters were superior grains, Small letters were inferior grains.

Microscopic observation revealed that both superior and inferior grains of soft and non-soft rice contained starch granules exhibiting an irregular polygonal shape (**Figure 2**). However, superior grains of soft rice displayed a higher degree of fragmentation (**Figures 2A, B**), whereas inferior grains of soft rice exhibited a greater abundance of pores within their starch granules (**Figures 2a, b**), in comparison to those of non-soft rice. These morphological differences in starch granules provide further evidence that the starch biosynthesis process is altered in soft rice, particularly within its inferior grains.

#### 3.4.2 Protein content and composition

Analysis of protein content and composition revealed significant differences between superior and inferior grains within both soft and non-soft rice (**Table 5**). Compared to non-soft rice, superior grains of soft rice exhibited higher levels of total protein (7.33%–8.57%), albumin (18.18%–29.03%), globulin (7.89%–48.48%), and glutelin (13.14%–17.39%), and a reduced prolamin content (2.27%–22.00%). Similarly, inferior grains of soft rice showed higher levels of total protein (7.10%–8.52%), albumin (7.50%–28.95%), globulin (12.20%–35.90%), and glutelin (13.49%–16.51%) compared with non-soft rice, and reduced prolamin content (4.26%–24.53%). Within soft rice, inferior grains displayed higher levels of total protein (4.00%–5.60%), albumin (7.50%–25.64%), globulin (6.52%–29.27%), prolamin (7.50%–15.38%), and glutelin (1.85%–5.52%) compared with their superior grains. Likewise, inferior grains of non-soft rice exhibited increased levels of total protein (4.45%–5.43%), albumin (15.15%–29.03%), globulin (2.63%–24.24%), prolamin (6.38%–20.45%), and glutelin (2.46%–5.35%) compared with their superior grains.

### 3.5 Pasting properties of flour

Analysis of rice flour pasting properties revealed significant differences between soft and non-soft rice (**Table 6**). Soft rice, in both superior and inferior grain fractions, exhibited distinct pasting characteristics compared to non-soft rice. Specifically, superior grains of soft rice displayed higher peak viscosity (PV), with values between 8.28% and 12.34%, and substantially higher breakdown viscosity (BD), with values between 49.07% and 71.93%, compared to non-soft rice. Conversely, superior grains of soft rice showed lower trough viscosity (TV), final viscosity (FV), setback viscosity (SB), and pasting temperature (P_temp_), with reductions of 4.15% to 7.55%, 12.49% to 13.62%, 258.53% to 261.88%, and 0.92% to 1.76%, respectively. Similar trends were observed in inferior grain fractions. Soft rice inferior grains exhibited higher PV, with values between 3.49% and 7.37%, and higher BD, with values between 8.82% and 39.55%, compared to their non-soft counterparts. Conversely, TV, FV, SB, and P_temp_ were lower in the inferior grains of soft rice varieties, with reductions of 3.79% to 7.70%, 19.99% to 21.59%, 201.95% to 207.73%, and 0.86% to 2.81%, respectively. Differences were also observed between superior and inferior grains within each variety. Inferior grains of soft rice showed lower PV (15.63%–18.61%), TV (2.81%–5.94%), FV (11.57%–13.34%), BD (17.01%–38.08%), and P_temp_ (0.53%–2.42%) compared to their superior grain counterparts. Similarly, inferior grains of non-soft rice exhibited lower PV (11.65%–14.92%), TV (2.32%–6.62%), FV (3.28%–4.53%), BD (2.18%–11.35%), and P_temp_ (0.53%–1.44%), while also exhibiting higher SB values (3.75%–27.97%).

**Table 6:** Differences in starch pasting properties between superior and inferior grains of soft rice and non-soft rice (%).

Cultivar	Grain position	Peak viscosity (cp)	Trough viscosity (cp)	Final viscosity (cp)	Breakdown viscosity (cp)	Setback viscosity (cp)	Pasting temperature(℃)
NJ5718	SG	2845.5±10.20a	1634.5±7.78cd	2347.0±7.07c	1105.5±24.75a	-422.5±37.48d	75.2±0.35c
	IG	2351.5±47.38c	1588.5±4.95de	2056.0±29.70d	861.0±25.15c	-327.5±13.44c	74.8±0.07d
NJ9108	SG	2787.0±28.28a	1649.5±24.35bcd	2372.5±24.75c	1037.5±36.06b	-464.5±17.68d	75.5±0.28c
	IG	2316.0±12.73cd	1551.5±24.75e	2075.5±24.55d	684.5±16.26de	-340.5±27.28c	73.6±0.46e
HD5	SG	2533.0±38.18b	1768.0±29.70a	2711.0±15.56ab	696.0±15.56d	261.0±18.38b	76.6±0.16a
	IG	2190.0±8.49e	1681.0±22.63bc	2594.0±20.21b	629.0±8.49ef	304.0±16.97ab	75.8±0.10bc
HJ5	SG	2574.0±22.23b	1721.0±19.80ab	2717.0±24.04a	643.0±4.24def	293.0±11.31ab	76.2±0.18ab
	IG	2238.0±35.36de	1651.0±29.50bcd	2622.0±15.56ab	617.0±15.56f	334.0±15.56a	75.5±0.10c
F-value	Type (T)	**	**	**	**	**	**
	Position (P)	**	**	**	**	**	**
	T×P	*	NS	**	**	*	NS

Values in the same column with different letters are significantly different (p < 0.05). SG, superior grains; IG, inferior grains. Type represents two types of soft rice and non-soft rice. Position represents the position of superior and inferior grains. NS, No significant correlation, *p<0.05, **p<0.01.

### 3.6 Correlation analysis of eating and appearance quality with starch, protein, and the pasting properties of rice flour

**Figure 3:**
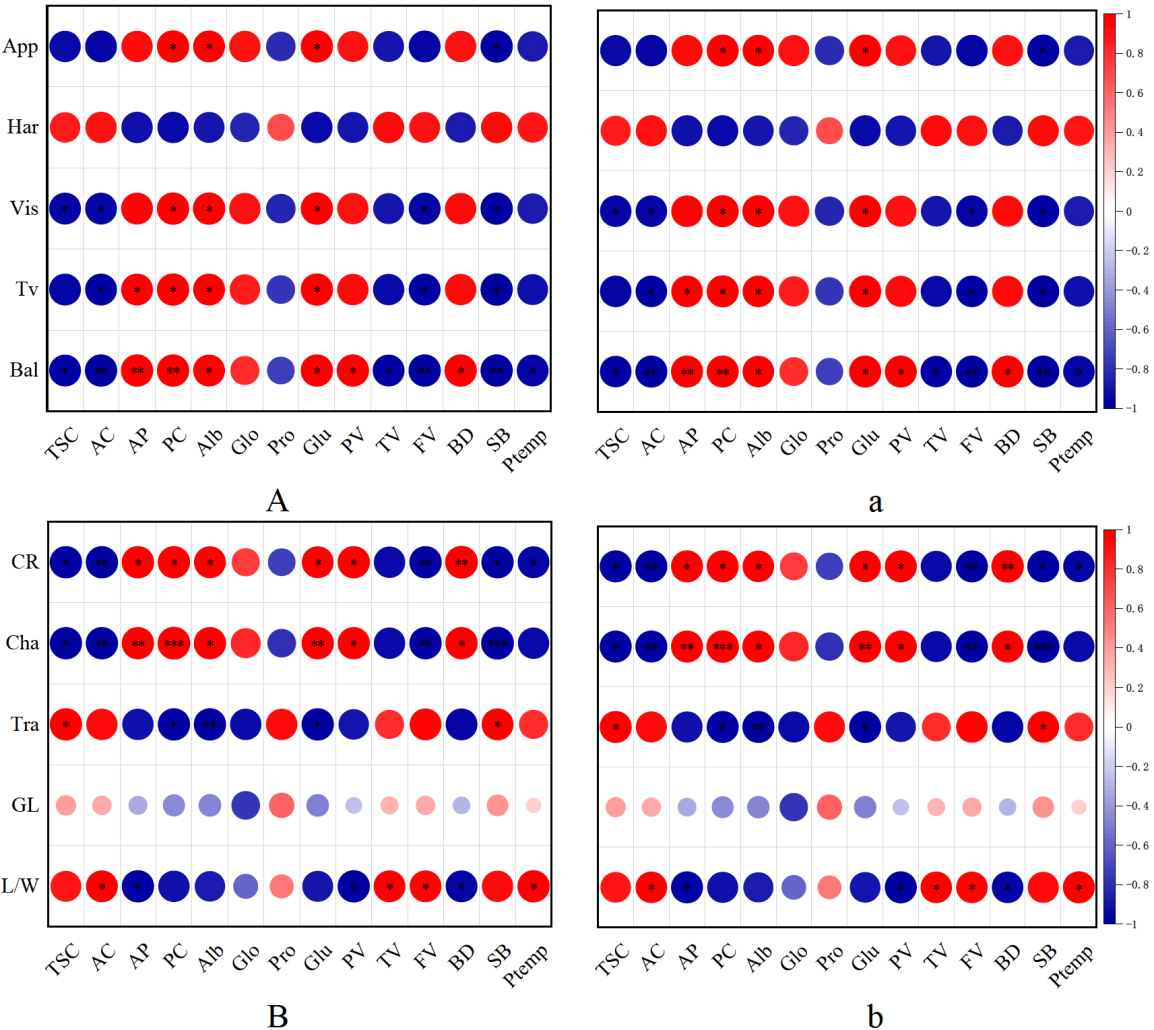
Correlation of eating quality and appearance quality of soft rice and non-soft rice with starch and protein content and pasting properties. **(A, a)** are the correlation analysis between eating quality and starch, protein components and pasting properties. **(B, b)** are t the correlation analysis between appearance quality and starch, protein components and pasting properties, Capital letters were superior grains, Small letter were inferior grains. TSC, Total starch content; AC, Amylose content; AP, Amylopectin content; PC, Protein content; Alb, Albumin; Glo, Globulin; Pro, Prolamin; Glu, Glutelin; CR, Chalky grain rate; Cha, Chalkiness degree; Tra, Transparency; GL, Grain length; L/W, Length/width ratio; App, Appearance; Har, Hardness; Vis, Viscocity; Bal, Balance; Tv, Taste value; BR, Brown rice rate; MR, Milled rice rate; HR, Head rice rate; PV, Peak viscosity; TV, Trough viscosity; FV, Final viscosity; BD, Breakdown viscosity; SB, Setback viscosity; Ptemp, Pasting temperature. *p<0.05, **p<0.01, ***p<0.001.

Correlation analysis revealed the relationship between rice taste, appearance quality and pasting properties of rice flour composed of starch and protein (**Figure 3**). The change trend of superior and inferior grains is basically the same, However, the difference in eating quality between the superior and inferior grains of soft rice was small, while the difference in appearance quality was large (**Table 6**, **Table 2**).

Regarding eating quality (**Figures 3A, a**), appearance, viscosity, balance and taste value also negatively correlate with total starch, amylose, prolamin, and pasting properties. Conversely, they positively correlate with amylopectin, total protein and its fractions, peak viscosity and breakdown viscosity. Notably, hardness displays an opposite trend compared to transparency and length/width ratio, it positively correlates with total starch, amylose, prolamin, and pasting properties, while negatively correlating with amylopectin, protein composition (albumin, globulin, and glutelin), peak viscosity and breakdown viscosity. In terms of appearance quality (**Figures 3A, a**), there exists a significant negative correlation between chalky grain rate and chalkiness degree, on the one hand, and total starch content, amylose content, prolamin, as well as pasting properties including trough viscosity, final viscosity, setback viscosity, and pasting temperature, on the other hand. Conversely, they exhibit a positive correlation with amylopectin content, total protein, and various protein composition (albumin, globulin, glutelin), as well as pasting properties such as peak viscosity and breakdown viscosity. Furthermore, transparency and length/width ratio, positively correlate with the aforementioned starch and prolamin components, as well as pasting properties (total starch, amylose, prolamin, trough viscosity, final viscosity, setback viscosity, and pasting temperature). In contrast, they negatively correlate with amylopectin, various protein contents (total protein, albumin, globulin, glutelin), peak viscosity and breakdown viscosity.

## 4 Discussion

### 4.1 Differences in rice quality between superior and inferior grains of soft and non-soft rice

In this study, the difference in processing quality between the two types of rice was minimal (**Table 1**). However, soft rice demonstrated superior eating quality compared to non-soft rice (**Table 4**), albeit exhibiting inferior appearance quality (**Table 2**). Given the significant effects of varietal diversity and grain position on quality characteristics (Zhang et al., 2021; Sun et al., 2022), we analyzed the quality of superior and inferior grains, respectively. In terms of appearance quality, the superior and inferior grains of soft rice showed higher chalky grain rate and chalkiness (**Table 2**), accompanied by a decrease in transparency. We believe that this decrease in transparency may be related to the morphological characteristics of rice grains, especially the fact that soft rice has a smaller length-width ratio (**Table 2**), resulting in a tendency towards a flattened cross-section. This flattened morphology reduces light penetration depth through the rice grains, thereby diminishing the clarity of internal structures and resulting in reduced grain transparency (Cao et al., 2024). In addition, the grain plumpness of soft rice was relatively low (**Table 3**), and the lack of plumpness further reduced the appearance quality of soft rice. When comparing superior and inferior grain varieties, significant differences are observed in soft rice, which are evident not only in apparent quality attributes but also in grain shape and plumpness characteristics. In particular, the high proportion of low-grain weight grains may be related to the poor development of inferior grains due to uneven nutrient distribution and insufficient light in soft rice (Zhang et al., 2021; Liu et al., 2024), which aggravates the decline of appearance quality of soft rice. Despite these deficiencies in appearance quality, the eating quality of soft rice was consistently superior to that of non-soft rice (**Table 4**). Both for superior and inferior grains, soft rice scored significantly higher in appearance, viscosity, balance, and taste value, and lower in hardness, compared to non-soft rice. Among the soft rice varieties, the inferior grains had slightly lower eating than the superior grains, indicating that the influence of inferior grains on the overall quality of soft rice was relatively small, which further demonstrated the stability of soft rice varieties in terms of eating quality. In conclusion, soft rice has outstanding advantages in eating quality despite its inferior appearance. This not only broadens our understanding of rice quality but also brings new ideas for breeding. In the pursuit of excellence in both appearance and eating quality, more attention should be paid to improving the appearance quality of soft rice varieties to meet market demand for high-quality rice.

### 4.2 Appearance quality, eating quality, and their relationship with starch and protein content and pasting properties in soft and non-soft rice

This study investigated the disparities in eating and appearance qualities between soft and non-soft rice. We further analyzed the relationship of these differences with starch and protein content and composition, as well as with pasting properties (**Figure 3**). The appearance quality of soft rice has been identified as a concern, as indicated by the appearance quality of soft rice is poor (**Table 2**). This issue arises primarily due to the low starch content in soft rice, which subsequently impacts the starch structure (Liu et al., 2017; Wei et al., 2018; Liu et al., 2024). Scanning electron micrographs reveal that starch granules in superior grains of soft rice exhibit polyhedral shrinkage accompanied by intergranular porosity (**Figures 1E, F**). Moreover, cracks are clearly visible on the cross-section of the endosperm (**Figures 1A, B**). These characteristics are even more pronounced in inferior granules, where starch granules tend to be spherical and significantly fragmented, accompanied by pore formation (**Figures 1e, f**; **Figures 2a, b**). The combined influence of these factors leads to multi-angle light scattering, ultimately resulting in chalkiness and a significant decline in the overall appearance quality of soft rice (Tao et al., 2019). Furthermore, the low amylose content in soft rice (**Table 5**) contributes to fewer water-binding sites and weaker hydrogen bonds within its endosperm. This results in a relatively loose binding state of water molecules, enhancing fluidity and increasing the likelihood of water loss through evaporation. As the starch matrix dehydrates due to dehydration and coagulation, the ordered structure becomes increasingly chaotic, leading to increased light scattering. Consequently, the particles become more opaque, and the endosperm appears darker (Zhu, 2022). Additionally, soft rice contains a higher content of globulin and glutelin (**Table 5**), resulting in more and larger protein bodies. These large protein bodies disrupt the tight accumulation of starch granules, creating gaps and irregular areas within the endosperm (Zhang, 2012; He et al., 2024). This disruption further exacerbates light scattering, causing the particles to exhibit chalkiness and reducing their transparency.

In terms of eating quality, although the high protein content of soft rice (**Table 4**) initially restricted the expansion of starch granules, the increased proportion of amylopectin and its unique long-branched structure facilitated the formation of a more viscous starch gel during the expansion process (Ma et al., 2022; Shi et al., 2023; Fan et al., 2023; He et al., 2024; Li and Gong, 2020). In addition, the high protein content also rendered the structural matrix relatively less firm and more susceptible to decomposition. Consequently, this limited yet rapid expansion effect, coupled with the weakening of the structural matrix, resulted in an increase in both peak viscosity and breakdown viscosity of the soft rice (**Table 5**). On the contrary, higher amylose content in non-soft rice (**Table 4**) interacts with proteins to form complexes during heating (Liu et al., 2020; Yin et al., 2023). This interaction hinders the decomposition of the starch structure, thereby reducing the leaching of starch from the granules (Shi et al., 2023; Yin et al., 2023). Consequently, non-soft rice exhibits higher hardness and lower viscosity characteristics, ultimately diminishing its eating quality (**Table 6**).

In summary, this study not only revealed significant differences in appearance and eating quality between soft and non-soft rice, but also deeply analyzed the complex relationship between these differences and starch and protein content and components. These findings provide a new perspective for improving the quality of rice in the future, helping to cultivate new rice varieties that have both excellent appearance and superior edible quality, in order to meet the market’s demand for high-quality rice.

## 5 Conclusion

The research results indicate that soft rice exhibits minimal differences in processing quality compared to non-soft rice but demonstrates significant advantages in eating quality across both superior and inferior grains. Specifically, soft rice displays higher appearance, viscosity, balance, taste values, and lower hardness. These superior eating qualities are partly attributed to the higher proportion of amylopectin in soft rice, which plays a significant role in enhancing the palatability of cooked rice. Notably, the quality difference between superior and inferior grains within soft rice is relatively small. However, there are certain deficiencies in the appearance quality of soft rice, mainly manifested as high chalky grain rates, high chalkiness degrees, low transparency, and a low proportion of high-specific-gravity grains. These defects are particularly prominent in inferior grains, resulting in greater differences in appearance quality between superior and inferior grains within the soft rice category. Additionally, due to the low amylose content and lack of hydrogen bonds, soft rice is easy to lose water, which further reduces the transparency of endosperm and aggravates the decline of its appearance quality. In this study, the high globulin and glutelin content of soft rice had larger protein bodies, which destroyed the close accumulation of starch granules, resulting in increased light scattering, chalkiness and reduced transparency.

## Data Availability

The original contributions presented in the study are included in the article/supplementary material. Further inquiries can be directed to the corresponding author/s.
